# Regional Differences in Penetration of the Protein Stabilizer Trimethoprim (TMP) in the Rat Central Nervous System

**DOI:** 10.3389/fnmol.2020.00167

**Published:** 2020-09-03

**Authors:** Benjamin V. Ineichen, Serena Di Palma, Endre Laczko, Shane A. Liddelow, Susanne Neumann, Martin E. Schwab, Alice C. Mosberger

**Affiliations:** ^1^Department of Health Sciences and Technology, Brain Research Institute, University of Zurich, ETH Zürich, Zurich, Switzerland; ^2^Functional Genomics Center Zurich, University of Zurich, ETH Zürich, Zurich, Switzerland; ^3^Neuroscience Institute, NYU School of Medicine, New York, NY, United States; ^4^Department of Neuroscience and Physiology, NYU School of Medicine, New York, NY, United States; ^5^Department of Ophthalmology, NYU School of Medicine, New York, NY, United States; ^6^Department of Clinical Neuroscience, Karolinska Institutet, Stockholm, Sweden

**Keywords:** trimethoprim, protein regulation, blood-brain barrier, blood-spinal cord barrier, central nervous system, pharmacokinetics, cerebrospinal fluid

## Abstract

Regulating gene expression at the protein level is becoming increasingly important for answering basic questions in neurobiology. Several techniques using destabilizing domains (DD) on transgenes, which can be activated or deactivated by specific drugs, have been developed to achieve this goal. A DD from bacterial dihydrofolate reductase bound and stabilized by trimethoprim (TMP) represents such a tool. To control transgenic protein levels in the brain, the DD-regulating drugs need to have sufficient penetration into the central nervous system (CNS). Yet, very limited information is available on TMP pharmacokinetics in the CNS following systemic injection. Here, we performed a pharmacokinetic study on the penetration of TMP into different CNS compartments in the rat. We used mass spectrometry to measure TMP concentrations in serum, cerebrospinal fluid (CSF) and tissue samples of different CNS regions upon intraperitoneal TMP injection. We show that TMP quickly (within 10 min) penetrates from serum to CSF through the blood-CSF barrier. TMP also shows quick penetration into brain tissue but concentrations were an order of magnitude lower compared to serum or CSF. TMP concentration in spinal cord was lower than in any other analyzed CNS area. Nevertheless, effective levels of TMP to stabilize DDs can be reached in the CNS with half-lives around 2 h. These data show that TMP has good and fast penetration properties into the CNS and is therefore a valuable ligand for precisely controlling protein expression in the CNS in rodents.

## Introduction

Temporal and spatial regulation of genes and proteins are key methods in modern biology and neuroscience. This field strongly relies on inducible gene expression through Cre-recombinase and Tet-ON/OFF systems (Bockamp et al., 2008). Many cellular processes in the brain, particularly during disease, are governed by protein regulation, modification, and transport, rather than gene expression changes (Vogel and Marcotte, 2012). However, only few methods exist to reliably regulate protein availability directly. The most promising techniques are using ligand-induced rescue from proteasomal degradation of a transgene-derived protein (Winter et al., 2015). In an early approach, a destabilizing domain (DD) modified from FK506- and rapamycin-binding protein (FKBP) fused to a protein of interest has been shown to lead to degradation by the proteasome, unless bound by the ligand Shield-1 (Banaszynski and Wandless, 2006; Banaszynski et al., 2006, 2008). To optimize this tool for use in the brain and spinal cord, a DD from bacterial dihydrofolate reductase (DHFR) which is bound and stabilized by the CNS permeable antibiotic trimethoprin (TMP, Tu et al., 1989) was developed. When fused to YFP and virally expressed in rat striatum, YFP protein levels can be controlled by TMP administered in drinking water (Iwamoto et al., 2010; Tai et al., 2012). This ligand-induced rescue from degradation has also been successfully achieved for a secreted protein (GDNF) (Quintino et al., 2013) and for Cre-recombinase in mice (Sando et al., 2013), and more recently with a constitutively active form of Akt kinase (Park et al., 2018). Most of these studies used peroral administration of TMP over several days or weeks. However, Sando et al. (2013) expressed DD-Cre under control of a synapsin promoter and showed fast induction of the transgene with TMP after intraperitoneal administration in mice, suggesting that systemic TMP reaches the brain within minutes to hours.

Fusing a DHFR-derived DD to a protein of interest and regulating its availability in the brain with systemic TMP administration therefore provides an interesting tool to achieve reversible and dose-dependent regulation of protein levels. Such methods are crucial to unravel the function of specific proteins in health and disease. However, to tightly control protein availability in the CNS, detailed pharmacokinetic data on penetration of TMP into specific brain areas are needed.

Despite TMP being listed by the World Health Organization as an essential medicine due to its antibiotic properties (World Health Organization, 2017) and many pharmacokinetic studies in humans (Barling and Selkon, 1978; Reeves and Wilkinson, 1979; Dudley et al., 1984; Bodilsen et al., 2018), only few pharmacokinetic studies in experimental animals exist (Tu et al., 1989) and none have looked at the temporal distribution in cerebrospinal fluid (CSF) and brain parenchyma from different CNS regions. TMP likely penetrates into specific CNS areas differently, due to regional differences in the blood-brain barrier (BBB) (Noumbissi et al., 2018), differences between BBB and blood-spinal cord barrier (Bartanusz et al., 2011), differences in penetration across the choroid plexus in different ventricles, or the CSF/perivascular flow (Ringstad et al., 2018) and/or biophysical properties of TMP that affect binding to specific tissue components.

We present here a pharmacokinetic study of TMP levels in different CNS compartments at early time points (10 min to 6 h) after intraperitoneal injection in rats. We aimed at answering two questions: (1) what is the temporal availability of TMP in blood, CSF and different CNS regions upon systemic application; and (2) are there differences for TMP penetration into different CNS regions?

## Materials and Methods

### Animals

Young adult, female Long Evans rats (*n* = 30; 230–350 g, 3–6 months of age, Charles River, Italy) were used in this study. Animals were housed in groups of two to four under a constant 12 h light/dark cycle with food and water *ad libitum*. To mitigate stress-related changes, all animals were handled over 4–5 days to familiarize them with researchers before any interventions. All *in vivo* experimental procedures were approved by the Veterinary Office of the Canton of Zurich, Switzerland. This study is written in accordance with the ARRIVE guidelines for reporting of animal studies (Kilkenny et al., 2010).

### Trimethoprim Application and Sample Collection

**FIGURE 1:**
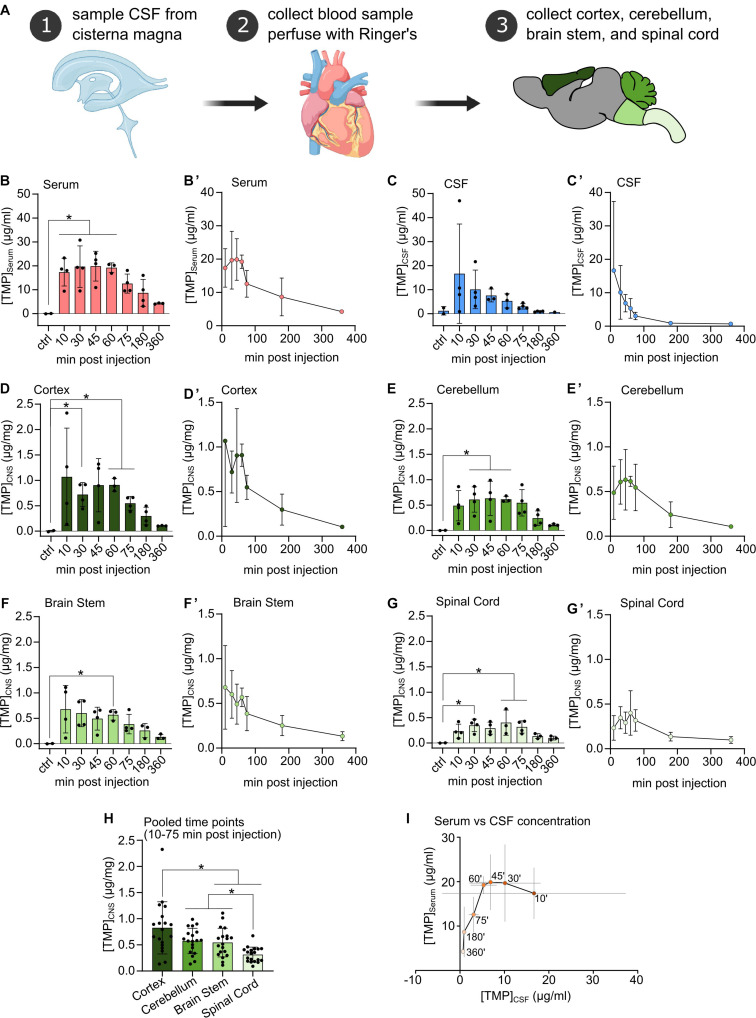
**(A)** Procedural schematic of sample collection after i.p. injection of Trimethoprim (TMP, 100 mg/kg body weight). **(B–G)** TMP concentrations detected in Serum, CSF, and freeze-dried CNS compartments at different time points after i.p. injection (100 mg/kg body weight) compared to vehicle injected controls. *N* = 3 or 4 per time point, *N* = 2 for controls, mean ± *SD*. Ordinary one-way ANOVA or Welch’s ANOVA if SD were significantly different, Dunnett’s multiple comparison correction, *p < 0.05. **(B’–G’)** Same data as in **(B–G)** plotted against time of sampling showing the concentration time course in each compartment. Mean ± *SD*. **(H)** CNS tissue samples pooled over the 10–75 min post-injection time points. Mean ± *SD*, *N* = 19, repeated measures one-way ANOVA, Tukey’s multiple comparison correction, *p < 0.05. **(I)** TMP concentration in the serum plotted against TMP concentration in the CSF across all sampling time points. Within the first hour after i.p. injection TMP concentration in the CSF decreased rapidly while serum levels remained relatively stable. Mean ± *SD* (gray lines), ‘ = minutes.

Trimethoprim lactate (Santa Cruz Biotechnology, #C1313) was dissolved in sterile, endotoxin-free injectable water at a concentration of 50 mg/ml and was stored in the dark and on ice during the whole experimental procedure. For all experimental procedures, rats were anaesthetized with 3–5% isoflurane. TMP was injected intraperitoneally (i.p.) at a dosage of 100 mg/kg body weight (Sando et al., 2013). Two rats were injected with injectable water without TMP and were immediately perfused, these rats served as controls. Rats were sacrificed at 10, 30, 45, 60, 75, 180, and 360 min post-injection (four rats per time point) (Figure 1A). Cerebrospinal fluid was sampled as previously described (Ineichen et al., 2017). Briefly, the cisterna magna was punctured dorsally through the skin using a 28 gauge cannula and CSF was checked visually for blood contamination (this method can detect down to 0.2% blood contamination; Habgood et al., 1992). Following an overdose of pentobarbital and reassurance of terminal anaesthesia, the thorax and abdomen of the rats were opened with scissors. The heart was punctured with scissors, and blood was collected to serum tubes. Rats were then transcardially perfused using 100 ml of ice-cold Ringer solution. Brains and spinal cords were quickly dissected under a stereomicroscope into cortex, cerebellum, brain stem, and spinal cord. Tissues were placed into light-shielded Eppendorf tubes and snap-frozen in liquid nitrogen.

Two rats were excluded from the study due to unsuccessful intraperitoneal TMP injection (one at time point 60 min and one at time point 360 min). Additionally, two CSF samples had to be excluded due to blood contamination.

### Tissue Extraction

CNS samples: all CNS samples were freeze-dried overnight, thus lowering the weight of the sample by 77–79% (Keep et al., 2012). Using a high-precision balance, 400 μg (± 5%) of each of the following CNS parts (freeze-dried) were placed in a light-shielded Eppendorf tube: cortex, brain stem, cerebellum and spinal cord. The tissue was homogenized using an electric pestle. Subsequently, 1.6 ml of ice-cold methanol was added to the tissue and vortexed for 1 min. Next, the samples were centrifuged at 16,000 g for 3 min at 4°C. The supernatant was then transferred to a fresh light-shielded Eppendorf tube.

CSF and serum samples: ice-cold methanol was added to CSF (4:1, 100 to 25 μl) and serum (4:1, 1000 to 250 μl) and samples were vortexed at low level for 1 min. Subsequently, samples were centrifuged at 3,000 g for 30 min at 4°C. The supernatant was transferred to a fresh light-shielded Eppendorf tube.

Before mass-spectrometry, the samples were stored at 4°C for 2 days in the dark.

### Mass Spectrometry

Samples were dried down using a gentle stream of nitrogen and reconstituted in an aqueous buffer containing 0.1% of formic acid (FA). The experimenter was blinded to respective sampling time points. Afterward, they were analyzed using a targeted approach by selected reaction monitoring (SRM) technology on a nano liquid chromatography-mass spectrometry (LC-MS) system (Eksigent NanoLC coupled to a Thermo TSQ-Vantage). Up to four transitions were selected for trimethoprim (291.16 > 123.13, 291.16 > 230.13, 291.16 > 261.13, and 291.16 > 275.15) with optimized collision energy, to ensure high selectivity and sensitivity for our targeted compound. The LC was set up in reverse phase mode, with a C18 column (Reprosil, 75 μm × 5 cm), a flow rate of 0.8 μL/min, and a gradient of 5 min. Buffer A was 0.1% FA and buffer B 0.1% FA in acetonitrile. Calibration curves and quality controls were included in the analysis for accurate quantification. The data was investigated by Xcalibur Quan Browser (Thermo) and concentration values calculated based on the area under curve (AUC) of the transition of highest signal intensity.

### Assessment of Pharmacokinetic Parameters and Pharmacokinetic Modeling

Clearance of TMP from the blood was estimated assuming a non-compartmental method (Tu et al., 1989) using formula (1):

(1) $$C\,l\,e\,a\,r\,a\,n\,c\,e=\frac{D}{A\,U\,C\,\left(S\,e\,r\,u\,m\right)}$$

Where D corresponds to the applied TMP dose [μg/g body weight] and AUC(Serum) equals the total area under the curve for serum concentration.

The volume of distribution (VD) was calculated using formula (2):

(2) $$V\,D\,\left(S\,e\,r\,u\,m\right)=\frac{D}{\left[C\right]\,S\,e\,r\,u\,m\,\left(0\right)}$$

Where the concentration in the serum at time of injection [C]Serum(0) is estimated by extrapolating from the early time points (10–45 min) using linear regression (Y = 0.07417^∗^X + 16.87, *R*^2^ = 0.87). [C]Serum(0) = 16.87 μg/ml.

The elimination constant K_*E*_ was calculated using formula (3):

(3) $$K_{E}=\frac{C\,l\,e\,a\,r\,a\,n\,c\,e}{V\,D}$$

Finally, partition coefficients (K_*p*_) (Hammarlund-Udenaes et al., 2008) for assessed CNS regions were estimated using formula (4):

(4) $$K_{p}=\frac{A\,U\,C\,\left(C\,N\,S\right)}{A\,U\,C\,\left(S\,e\,r\,u\,m\right)}$$

K_*p*_ describes the drug concentration present in the CNS compared to that in blood, used as a measurement of CNS penetration. The K_*p*_ is taken as an approximation to the as an approximation to the VD (Hammarlund-Udenaes et al., 2008).

Next, we applied the blood-brain exchange model proposed by Bradbury and Kleeman (1967) and later adapted by Blasberg et al. (1983), Patlak et al. (1983), Kakee et al. (1996), and Bickel (2005), to probe whether there is a unidirectional uptake phase for TMP into the CNS. During unidirectional uptake, the relationship between serum and CNS TMP concentration is predicted to follow the linear relationship (formula 5):

(5) $$\frac{\left[C\right]\,C\,N\,S\,\left(T\right)}{\left[C\right]\,S\,e\,r\,u\,m\,\left(T\right)}=K_{i\,n}^{*}\,\frac{AUC\left(0\to T\right)\left(Serum\right)}{\left[C\right]\,S\,e\,r\,u\,m\,\left(T\right)}+V\,i$$

Where [C]CNS(T) is the concentration in the corresponding CNS region (cortex, cerebellum, brain stem, or spinal cord) and [C]Serum(T) the concentration in the serum at time point T and AUC corresponds to the area under the curve for TMP in serum from time point 0 to T. Plotting $\frac{\left[C\right]\,C\,N\,S\,\left(T\right)}{\left[C\right]\,S\,e\,r\,u\,m\,\left(T\right)}$ against $\frac{A\,U\,C\,0\to T\,\left(S\,e\,r\,u\,m\right)}{\left[C\right]\,S\,e\,r\,u\,m\,\left(T\right)}$, K_*in*_ (the unidirectional influx constant) and V_*i*_ (the initial volume of distribution) can be estimated by fitting a linear regression model. We assessed unidirectional uptake for time points 10–75 min when linearity was strongest.

To assess the half-life of TMP in different CNS compartments, and in CSF, one-phase exponential decay functions were fitted to the concentration time curves. For compartments with significant unidirectional uptake over the first 75 min, and the serum samples themselves, the decay curve was fitted starting from sample *T* = 60 min. For the other compartments the decay curve was fitted starting from sample *T* = 30 min, as the first sample at 10 min showed high variability (see Figure 1).

Peak concentrations were formally defined as the highest mean value concentrations of TMP in the corresponding tissue/fluid compartment.

### Statistical Analyses

All animals were randomly allocated to respective time points. For comparison of CNS regions, a repeated measures one-way ANOVA and Tukey’s multiple comparison correction was performed. Ordinary one-way ANOVA or Welch’s ANOVA, if standard deviations (*SD*) were significantly different, were performed on time course data with Dunnett’s multiple comparison correction. Data from bar plots and data described in the text are shown as mean ± *SD*. Asterisks indicate statistical significance: ^∗^*p* < 0.05. To test for unidirectional uptake, a correlation analysis was performed. All statistical analyses and linear and non-linear curve fitting was performed with GraphPad Prism Version 8.4.2.

## Results

### Uptake of TMP Into the CSF and CNS

Upon intraperitoneal injection, TMP quickly appeared in the blood at high concentrations (∼17 μg/ml) at the earliest time point (10 min post-injection), plateaued at this level for up to 60 min, and subsequently decreased in concentration (Figures 1B,B’). TMP also penetrated quickly into the CSF compartment, where it reached a similar level as in the serum at 10 min (∼16 μg/ml), but with a high variability among samples. Subsequently, CSF TMP concentrations decreased rapidly (Figures 1C,C’) even during the period of stable initial serum TMP concentration (at 10–60 min, Figure 1I).

In the different CNS tissue compartments, TMP levels were high at 10 min, and sustained up to 60 min. before falling off (Figures 1D,D’–G,G’). Similar to blood, TMP levels plateaued in all CNS regions between these time points. Of note, compared to serum and CSF, TMP levels in the different CNS tissues were at least an order of magnitude lower (Figures 1D,D’–G,G’). The lower availability of TMP in brain tissue is also emphasized by the considerably smaller area under the curve (AUC) for TMP in the individual CNS regions compared to serum and CSF (Table 1). Trimethoprim remained detectable at 360 min post-injection in all analyzed CNS regions, CSF and serum, albeit at rather low levels [around 21% for serum (compared to peak concentration), 4% for CSF and 10–17% for the different brain regions]. In the myelin-rich spinal cord and brain stem, 20–25% of the peak concentration was still detectable at 360 min post-injection (Table 1). To compare the overall availability of TMP in different CNS regions, we pooled all samples from 10 to 75 min post-injection for each area (Figure 1H). Overall TMP availability in the cortex (mean ± *SD*: 0.83 ± 0.5 μg/mg) was significantly higher than in the brain stem (mean ± *SD*: 0.54 ± 0.27 μg/mg, *p* = 0.004) and spinal cord (mean ± *SD*: 0.31 ± 0.14 μg/mg, *p* = 0.001). TMP concentration was the lowest in the spinal cord, significantly below the concentration in the brain stem and cerebellum (mean ± *SD*: 0.58 ± 0.24 μg/mg, *p* = 0.003 and *p* < 0.0001, respectively).

**TABLE 1:** Areas under the curve (AUC) and peak concentrations for TMP in serum, CSF and different CNS regions.

Tissue	AUC [μg*min* ml^–1^;mg^–1^]	[C] peak [μg/ml;mg]	[C] peak (time)	% [C] endpoint (6 h) compared to [C] peak
Serum	3,563	19.875	45 min	21
CSF	992	16.645	10 min	4
Cortex	141	1.068	10 min	10
Cerebellum	113	0.631	45 min	17
Brain stem	108	0.679	10 min	20
Spinal cord	67	0.400	60 min	24

*TMP concentration was much higher in serum and CSF compared to CNS. Endpoint = 6 h post-injection. Peak concentrations are formally defined as the highest mean value concentrations of TMP in the corresponding tissue/fluid compartment.*

### Pharmacokinetic Modeling

**FIGURE 2:**
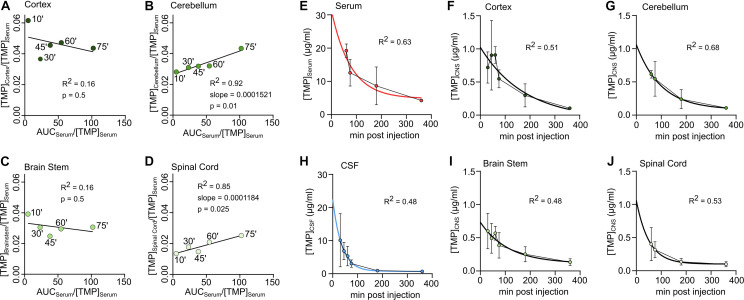
Unidirectional uptake modeling in cortex **(A)**, cerebellum **(B)**, brain stem **(C)** or spinal cord **(D)**. Linear regression lines fitted to the first 5 sample time points (‘ = minutes). For good fits with significantly non-zero slope the slope is reported as ml *mg^–1^ *min^–1^. **(E–J)** Concentration-time data in different tissues during decay. An exponential decay function was fitted to determine the half-life of TMP. **(E)** Serum, **(F)** Cortex, **(G)** Cerebellum, **(H)** CSF, **(I)** Brain stem, **(J)** Spinal Cord.

To estimate whether TMP penetrated the blood-brain/blood-spinal cord barrier unidirectionally, $\frac{\left[C\right]\,C\,N\,S\,\left(T\right)}{\left[C\right]\,S\,e\,r\,u\,m\,\left(T\right)}$ was plotted against $\frac{A\,U\,C\,0\to T\,\left(S\,e\,r\,u\,m\right)}{\left[C\right]\,S\,e\,r\,u\,m\,\left(T\right)}$ for the period during which serum levels were stable (10–75 min) (Figures 2A–D) and a linear regression line was fitted to the data. The linear fit was poor for cortex (Figure 2A) and brain stem (Figure 2C) compartments but a good linear fit was achieved for cerebellum (Figure 2B) and spinal cord (Figure 2D). According to the model by Bradbury and Kleeman (1967), the slope of the regression line was used to estimate the K_*in*_ (unidirectional influx constant) for the cerebellum (K_*in*_ = 0.1512 ml ^∗^ g^–1^^∗^ min^–1^) and spinal cord (K_*in*_ = 0.1184 ml ^∗^ g^–1^^∗^ min^–1^). These influx constants are relatively low considering the lipophilic nature of TMP, and were only found in two of the four brain areas. Therefore, the main uptake of TMP into the CNS is most likely bidirectional and diffusion driven.

To use TMP to control the levels of a protein of interest in the rodent CNS, an important factor is the duration of availability in different tissues. For the serum and CNS compartments, basic pharmacokinetic parameters were calculated (see section “Materials and Methods”) and were summarized in Table 2. The partition coefficient (K_*p*_) (a proxy for the volume of distribution in the CNS) was highest for the cortex (∼0.04) and lowest for the spinal cord (∼0.02).

**TABLE 2:** Pharmacokinetic parameters for trimethoprim for serum, cerebrospinal fluid (CSF) and different central nervous system tissues.

Parameter	Serum	CSF	Cortex	Cerebellum	Brain stem	Spinal cord	Unit
Clearance	28	–	–	–	–	–	ml*kg^–1^*min^–1^
Volume of distribution	5,928	–	–	–	–	–	ml*kg^–1^
Elimination constant (K_*E*_)	0.0047	–	–	–	–	–	min^–1^
Half-life (T_0_._5_)	114	55	154	129	109	95	min
Partition coefficient (K_*p*_)	–	0.2784	0.0394	0.0318	0.0302	0.0188	–

*For Serum clearance, volume of distribution and K_*E*_ were calculated from formulas (1), (2), and (3), respectively. For CSF and CNS tissues K_*p*_ calculated from formula (4). All Half-lives estimated by fitting an exponential decay function (Figures 2E–J).*

To estimate the half-life of TMP in different compartments, a one-phase exponential decay was fitted to the concentration-time data for the time points of decay (see section “Materials and Methods,” Figures 2E–J and Table 2). As mentioned above, the half-life of TMP in the blood (114 min) was about twice as long as in the CSF (55 min). In the CNS, TMP showed an anterior-posterior gradient for the half-life parameter, with cortex having the longest half-life (154 min) and spinal cord the shortest (95 min). This gradient is also evident in the overall concentration over the first 75 min after injection (Figure 1H) which was highest for cortex and lowest for spinal cord.

## Discussion

Methods to manipulate levels of a given protein in the CNS are important tools to study the role of specific proteins in health and disease. A recent promising technique to regulate protein levels in CNS tissue *in vivo* uses a destabilizing domain fused to a protein of interest which leads to degradation of this protein unless it is bound by TMP (Cho et al., 2013). TMP has been shown to reach the brain in short time following i.p. injection (Sando et al., 2013), an important prerequisite for effective use in neuroscience. Here we provide a detailed pharmacokinetic study describing the concentration of TMP in serum, CSF, and different CNS regions in rats after a high intraperitoneal TMP dose. We show that TMP reaches the CSF from the blood in a short time (10 min). Interestingly, while blood levels remained at a plateau for 1 h before starting to decay, CSF TMP levels fell off earlier (30 min) and more steeply. TMP also readily penetrated most CNS areas within an hour, but concentrations were an order of magnitude lower compared to serum or initial CSF – indicative of either slow penetration, or active exclusion by the BBB or the blood-CSF barrier. Finally, we found that TMP penetrates the spinal cord the least and cortex the most, with differences in retention levels – suggesting regional differences in exposure (e.g., from the blood or CSF) that could be due to differences in blood vasculature or cellular composition of the parenchyma.

Our data show early detection of TMP in CSF (sampled from the cisterna magna) at concentrations similar to serum. This indicates a rapid penetration of TMP through the blood-CSF barrier in rat. This finding is in line with findings in humans showing high TMP levels in CSF upon systemic application (Dudley et al., 1984). Interestingly, however, TMP levels in CSF peaked 10 min after i.p. injection and decreased by 30 min, in contrast to serum levels which remained high up to 60 min. The longer and higher retention rate in plasma could be due to high plasma protein binding which has been shown being around 65–70% in rodents (mainly to albumin) (Berneis and Boguth, 1976). TMP levels will also likely remain high in the blood plasma due to continued release from the injection site and circulation, while in the CSF levels dropped as the drug penetrated into the CNS tissue, and CSF is rapidly cleared into the systemic circulation from the intrathecal compartments. An alternative would be a downregulation of TMP transport mechanisms at the level of the choroid plexus epithelium that comprise the blood-CSF barrier. This has been reported for some other drug transporters (e.g., MDR1, OCT1, OATP) following drug administration (Dresser et al., 2000; Kubota et al., 2006).

A prior TMP pharmacokinetic study in rats found the highest TMP levels among different tissues in the kidneys (Tu et al., 1989) in which organic cation transport (OCT) proteins were shown to transport TMP (Ahlin et al., 2008). TMP has been reported to inhibit OCT2, limiting drug secretion (Ferrazzini et al., 1990; Grün et al., 2013). OCT expression has also been reported in the brain, particularly in the brain microvasculature (Amphoux et al., 2006; Lin et al., 2010). Yet, OCTs were not detected in the brain in a recent transcriptomic database of the mouse BBB under healthy conditions (Munji et al., 2019), but interestingly, the expression increased in chronic inflammatory states. As OCT also transports neurotransmitters (Lee et al., 2001), the inhibition or up/down-regulation of OCTs by TMP may have off-target effects that should be taken into consideration while using a TMP DD approach. To control for this, we recommend to include “TMP only” controls in any experimental design.

TMP in CNS tissue could come from (1) the CSF via the perivascular spaces, which surround blood vessels penetrating the CNS, (2) penetration from the CSF directly into the brain parenchyma lining the ventricles, and/or (3) the systemic circulation by crossing the BBB. Regarding (1): some solutes such as gadolinium contrast-agent are efficiently distributed within the brain through the perivascular spaces, reaching deeper brain structures within 50–100 min post-application (Iliff et al., 2013). Interestingly, TMP levels plateaued for around 45–60 min after application in other areas in spite of the decreasing CSF levels. Slow convective distribution of TMP via transparenchymal flux is a possible, but unlikely reason for this plateau (Louveau et al., 2017; Ringstad et al., 2018). (2) seems unlikely due to the rapid depletion of TMP from the CSF, presumably by CSF convective flow back to the blood; yet, the cortical – spinal cord TMP gradient would be in line with TMP distribution along the natural CSF flow routes along the ventricles and spinal cord central canal (Ma et al., 2019). (3): systemic TMP entering from the capillary bed over the BBB is another source of CNS TMP. Our data indicate a relatively low penetration of TMP through the BBB which is in line with an earlier pharmacokinetic study of TMP in rats which compared whole brain samples with other organs (Tu et al., 1989). We found a small unidirectional uptake constant for cerebellum and spinal cord regions, but no indication of unidirectional uptake in cortex or brain stem. Whereas organ-specific transporter system for TMP have been described, including the cationic active transport in the kidneys (Cacini and Myre, 1985), no such active transport systems have been described in the CNS so far. Thus, a passive bidirectional diffusion of TMP, a small lipophilic molecule, through the BBB seems most likely. It is possible, however, that specific transporters for TMP may be heterogeneously present in different regions of the BBB (Ueno et al., 2000; Suzuki et al., 2003; Nyul-Toth et al., 2016).

It is worth noting that the brain vasculature and therefore the BBB has been shown to be affected by sex hormones (Öztaş et al., 2000; Dan et al., 2003; Krause et al., 2006). Thus, differences between sexes may exist in the distribution of TMP in the CNS. Additionally, age affects the BBB and brain vasculature (Hafezi-Moghadam et al., 2007; Blau et al., 2012). Experiments conducted in very young or aged rodents may see different results for TMP distribution than described here.

We found an anterior-posterior gradient in overall TMP concentration, volume of distribution (K_*p*_), and half-life from cortex, cerebellum and brain stem, to spinal cord. TMP penetrated best into the cortex, where it remained for longer, and least into spinal cord. There are several potential underlying mechanisms: spinal CSF flow is lower compared to cranial CSF flow, as this has been shown in sheep where it has been estimated to be around 25% (Bozanovic-Sosic et al., 2001), a similar ratio is assumed in mice (Ma et al., 2019). Second, the blood-spinal cord barrier shows distinct morphological and functional features (reviewed in Bartanusz et al., 2011) which could lead to increased removal from the spinal cord. On the other hand, hydrophobic interactions of TMP with the myelin- and lipid-rich spinal cord tissue could lead to a slow accumulation and delayed wash-out of the drug, as indicated by the higher retention rate observed at 360 min.

Additional CNS regions, such as striatum and hippocampus, may follow this antero-posterior gradient depending on their vasculature make up and CSF access. Sufficient bioavailability of TMP for striatum has been previously reported in two different studies using chronic peroral TMP administration (Tai et al., 2012; Cederfjäll et al., 2015).

Most TMP is largely removed from CSF and all measured brain regions after 360 min. This is in line with previous pharmacokinetic data in rats (Tu et al., 1989).

The plateau and/or peak TMP concentration are a key aspect in the context of TMP as a DD inactivating drug; a minimal concentration is required for efficient stabilization of a target DD-fused to a protein of interest. During the plateau phase in the CNS, TMP concentrations ranged between 500 and 1000 ng/mg for our analyzed brain regions and between 250 and 500 ng/mg for the spinal cord, as measured by the highly sensitive liquid chromatography-mass spectrometry (Tautenhahn et al., 2008). *In vitro*, DD fused to the N terminus of YFP was fully stabilized by 1 μM TMP, corresponding to 290 ng/ml or 0.29 ng/mg (Iwamoto et al., 2010). This shows that with an i.p. dose of 100 mg/kg body weight in rats, effective levels of TMP well above 1 μM can be reached in all parts of the CNS. Of note, despite measuring drug concentrations in freeze-dried CNS tissue – thus overestimating the true tissue concentration by a factor of approximately 5 (assuming 80% brain water content; Keep et al., 2012) – effective local TMP levels are reached. Therefore, these findings support the notion that significantly smaller systemically applied TMP doses could be sufficient to reach effective CNS TMP levels.

The rapid penetration of TMP into the CNS compartments 10 min after i.p. injection observed in the present study shows that TMP has a favorable pharmacokinetic profile as a ligand to stabilize DD-coupled target proteins within the CNS. The rather short half-life of TMP is an additional, important advantage for precisely controlling protein expression windows. Care should be taken when administering TMP on longer time-scales, however, due to its multiple systemic adverse effects including bone-marrow toxicity (Nau et al., 2010) and potential neurological adverse effects such as meningitis (Ho and Juurlink, 2011). In conclusion, our results show a favorable pharmacokinetic profile of TMP as a ligand for DD mediated protein expression in the CNS with regional differences pointing toward an antero-posterior gradient of distribution.

## Data Availability Statement

The raw data supporting the conclusions of this article will be made available by the authors, without undue reservation, to any qualified researcher.

## Ethics Statement

The animal study was reviewed and approved by the Veterinäramt Kanton Zürich, Switzerland.

## Author Contributions

BVI and ACM conceived, designed, and executed the experiments, and did the pharmacokinetic modeling. MES, SAL, SD, and EL contributed to the experimental design. SD and EL performed the mass spectrometry. BVI, ACM, and MES wrote the manuscript and prepared the figures. All authors provided critical input on the manuscript.

## Conflict of Interest

The authors declare that the research was conducted in the absence of any commercial or financial relationships that could be construed as a potential conflict of interest.
